# "Hands Up" and Social Distancing: A Rare Case of Bilateral Luxatio Erecta During the Second Wave of the COVID-19 Pandemic Lockdown Period

**DOI:** 10.7759/cureus.31675

**Published:** 2022-11-19

**Authors:** Dimitrios Ntourantonis, Ioanna Lianou, Anastasia Ampariotou, Vasileios Daskalopoulos

**Affiliations:** 1 Emergency Department, General University Hospital of Patras, Patras, GRC; 2 Orthopedics and Traumatology, General University Hospital of Patras, Patras, GRC; 3 Orthopedics and Traumatology, General University Hospital of Patras, Patra, GRC

**Keywords:** emergency department, bilateral, luxatio erecta, inferior glenohumeral dislocation, inferior shoulder dislocation

## Abstract

In this paper, we present an interesting and very rare case of bilateral luxatio erecta as a result of unattended at-home sports activities during the lockdown period due to the second COVID-19 pandemic wave.

A 31-year-old man presented to the emergency department (ED) with the characteristic "hands up" position after an injury on both shoulders while he was performing unsupervised weightlifting training at home during the lockdown.

After the successful reductions, the neurovascular status for both upper extremities was evaluated and confirmed without impairment. The patient has fully recovered and has gained full range of motion on both shoulders without any signs of instability.

Luxatio erecta is a low-incidence injury, while the presence of this injury in both upper extremities is thought to be extremely rare, with only a few cases published in the literature to date. Bilateral cases are associated with a high rate of complications. ED physicians should maintain an increased awareness for prompt recognition, particularly in polytrauma patients, as the presence of this injury increases the complexity of managing this type of patient in the ED due to the abducted arms.

## Introduction

The COVID-19 pandemic has dramatically changed our lives during the last three years. Forced, severe, and long-lasting restrictions caused major problems and reduced access to various services and activities, and the loss of direct social interactions. Millions of people started to work from home, losing their daily interaction with other individuals and, furthermore, changing their daily diet habits. As a result, weight changes occurred, as expected. All these changes led to a distinct change in the way people spend their time, forcing them to find new ways of social interactions and new training habits. On the other hand, the impact of trauma cases at the emergency department (ED) decreased during the lockdown periods, as it is directly linked to reduced activities outside the home. In this report, we present a very rare case of traumatic bilateral luxatio erecta (TBLE) as a result of unattended at-home sports activities during the pandemic. The diagnostic algorithm, the treatment protocol, and the reduction technique are analyzed, emphasizing the prompt diagnosis and careful treatment performed at the ED.

Historically, shoulder dislocation is thought to be the most commonly treated dislocation in the emergency department in adults. However, inferior dislocations, known as luxatio erecta (LE), compromise only 0.5% of all shoulder dislocations, compared to anterior and posterior ones, which account for 95-97% and 2-4%, respectively [1-3]. The first case of LE was described by Middledorpf and Scharm in 1859 [4], and it was unilateral. Because of its uncommon occurrence, LE is often misdiagnosed as an anterior dislocation. LE is often accompanied by neurovascular injuries, including neuropraxia of the brachial plexus, ulnar or radial nerve, axillary artery injury, or upper limb deep vein injury. Around 31% of all patients with mololateral LE present with neurological deficits, and 9% of them with vascular compromise, while at least 60% of bilateral cases present with neurovascular deficits [5]. Furthermore, musculoskeletal injuries, including disruption of the supraspinatus, infraspinatus, subscapularis, and pectoralis major and/or fractures of the clavicle, coracoid, acromion, inferior glenoid, and greater tuberosity of the humerus are common after this type of dislocation. When ongoing pain or instability symptoms are suspected after initial management, MRI scanning should be performed to demonstrate rotator cuff or labral injury. Finally, emergency physicians should have a high index of suspicion when evaluating patients with similar mechanisms of injury.

The first case of TBLE was described by Murard [6] in 1920, and since then, only a few cases have been published in the medical literature.

## Case presentation

A 31-year-old man presented to the emergency department after an injury on both shoulders while he was performing unsupervised weightlifting training during the lockdown period due to the second wave of the COVID-19 pandemic. The patient was working from home as a programmer during this period and was trying to improve his physical status. He was not a competitive athlete, and he had never in the past trained in weightlifting. He has no other remarkable medical history or major injury on the upper limb before this. The exercise that caused the injury was described as an overhead lifting of two custom-made 25-kilogram concrete plates. We suppose that both arms were forced in hyperabduction stress, while the humeral neck was directed superiorly against the acromion process and the head out of the glenoid fossa inferiorly, as it has been described by Frank et al. [7]. 

**Figure 1:**
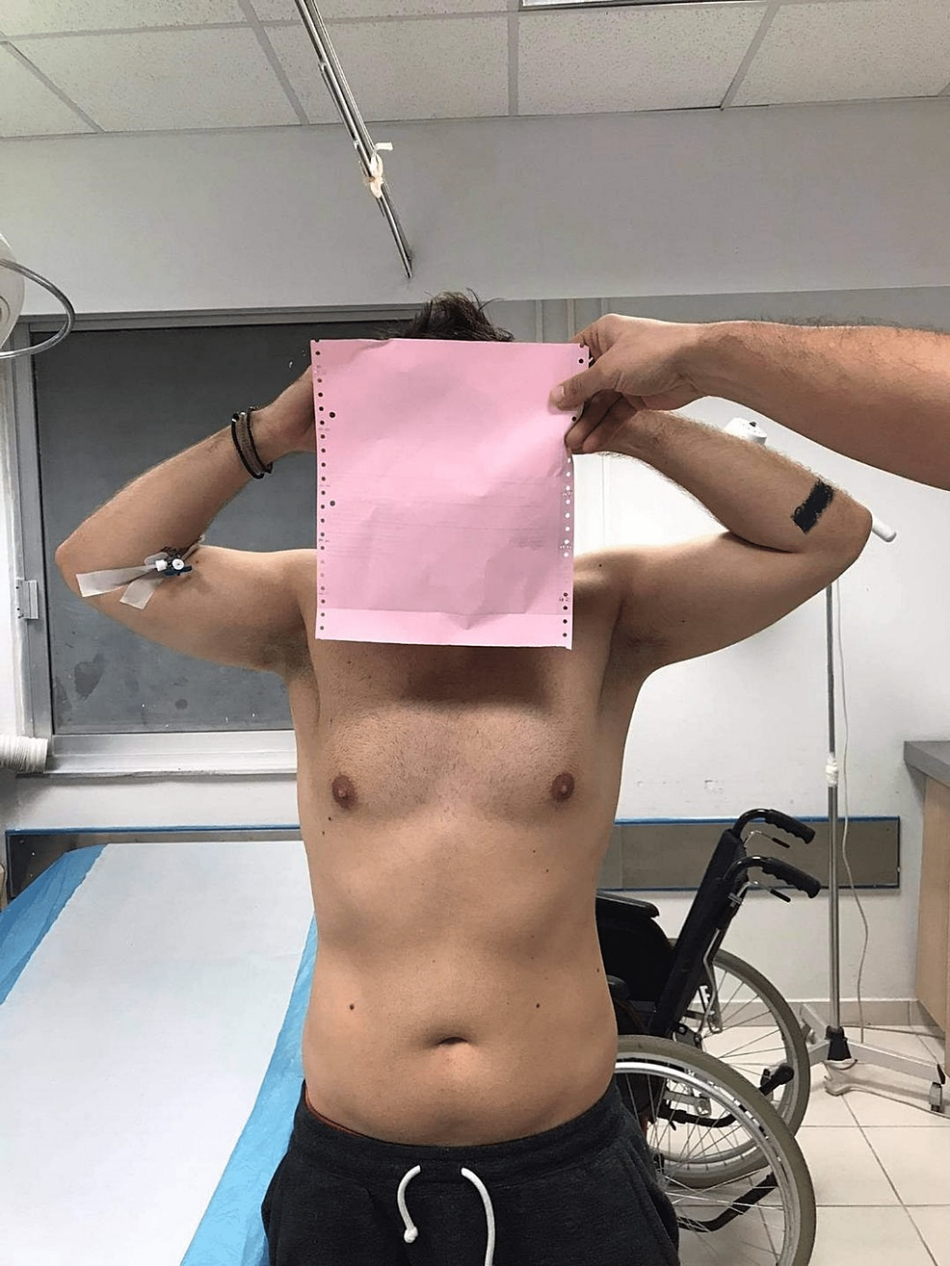
Patient presentation at the emergency department

**Figure 2:**
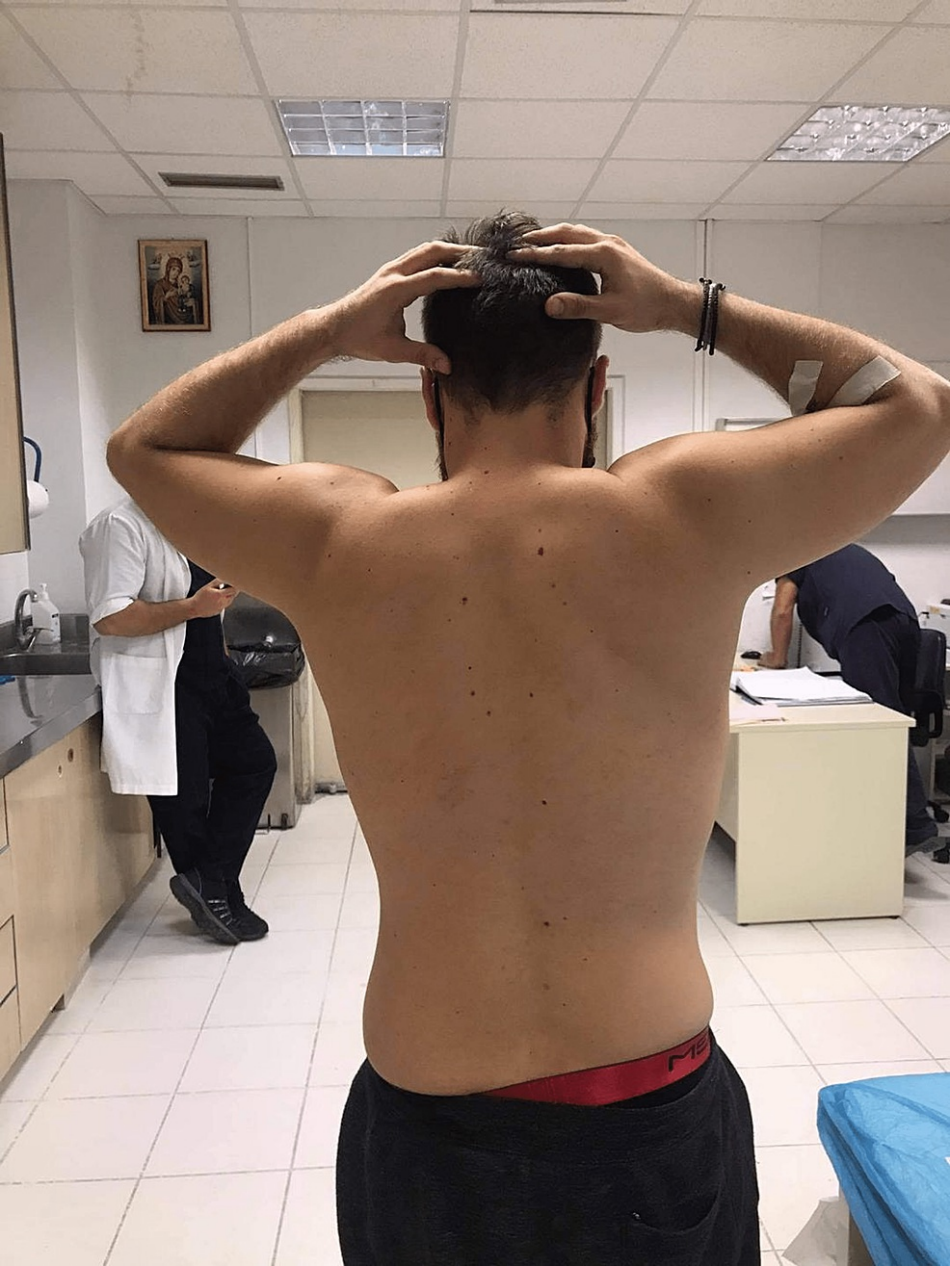
Patient presentation at the emergency department

On presentation to the emergency department, the patient was complaining of intense pain in both shoulders with the inability to adduct the arms due to mechanical resistance and pain. Both upper limbs had the typical "hands up" position (abducted shoulder, flexed elbow, and pronated forearm) while both hands were held behind his head (Figures 1, 2).

Any attempt at passive movement of the shoulders was causing extreme pain and resistance. Physical examination revealed an alert man without reported loss of consciousness. No other injuries were reported, and no deformities of the elbow, forearm, or wrist were noticed. He denied any paresthesia or sensory deficits during the initial examination. No vascular deficits had been detected. Bilateral LE was suspected by the physicians of the ED, according to the mechanism of injury and a clinical presentation consistent with this type of injury.

**Figure 3:**
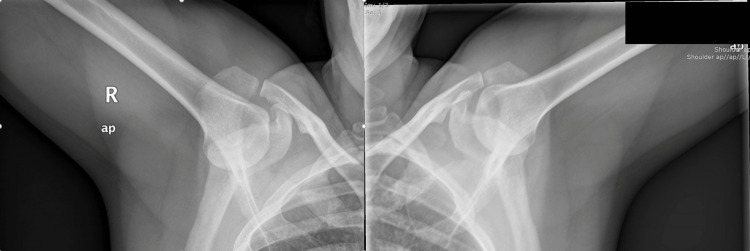
Anteroposterior X-ray showing a bilateral inferior glenohumeral dislocation

Plain anteroposterior (AP) radiographs of both shoulders confirmed the initial diagnosis (inferior subluxation of both humeral heads), with no obvious fracture of the head or the glenoid fossa (Figure 3).

A treatment plan was carefully organized as the increased incidence of neurovascular injuries relative to other shoulder dislocations required an extremely careful approach.

Multimodal analgesia was admitted to the patient. Particularly, 5mg of diazepam, 1g of paracetamol, and 25mg of dexketoprofen tromethamine were given intravenous combined with an intramuscular dose of 50mg of pethidine. Once the patient was in adequate analgesia, a two-step reduction technique [8] was performed by the ED physicians. Firstly, the inferior dislocation of the right upper extremity was reduced to an anterior one, with applied traction in the direction of the longitudinal axis of the arm (the elbow was supported, and the limb was pulled upward and outward). Another person of the team applied counter traction by stabilizing the torso. While this traction-counter traction was maintained, the arm was adducted, and when it was between 90-45 degrees from the body, an audible pop confirmed the conversion to anterior dislocation. This transformation was evaluated by common clinical signs of anterior dislocation, such as loss of the normal contour of the glenoid and a prominent acromion posteriorly and laterally. The anterior dislocation was then successfully reduced with Kocher's maneuver [9]. The same technique was performed on the contralateral side. After the successful reductions, the neurovascular status for both upper extremities was evaluated and confirmed without impairment. Both sides were immobilized with protective slings.

**Figure 4:**
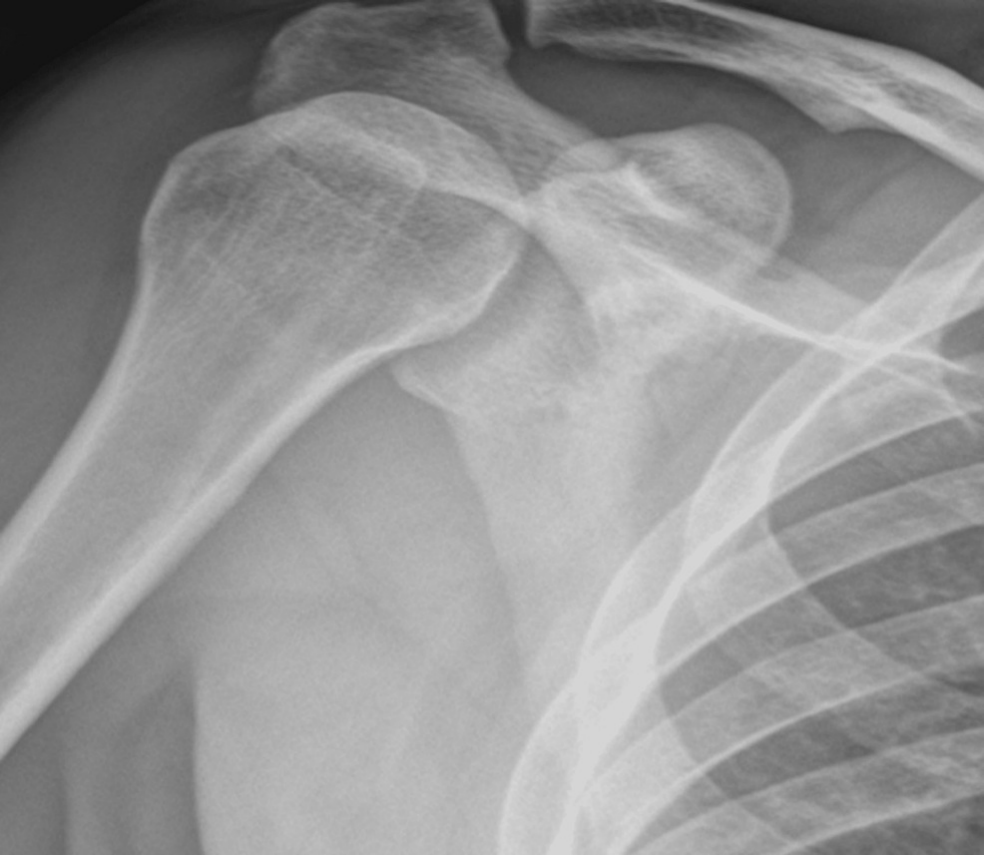
Anteroposterior X-ray of the right shoulder after close reduction by two-step technique maneuver

**Figure 5:**
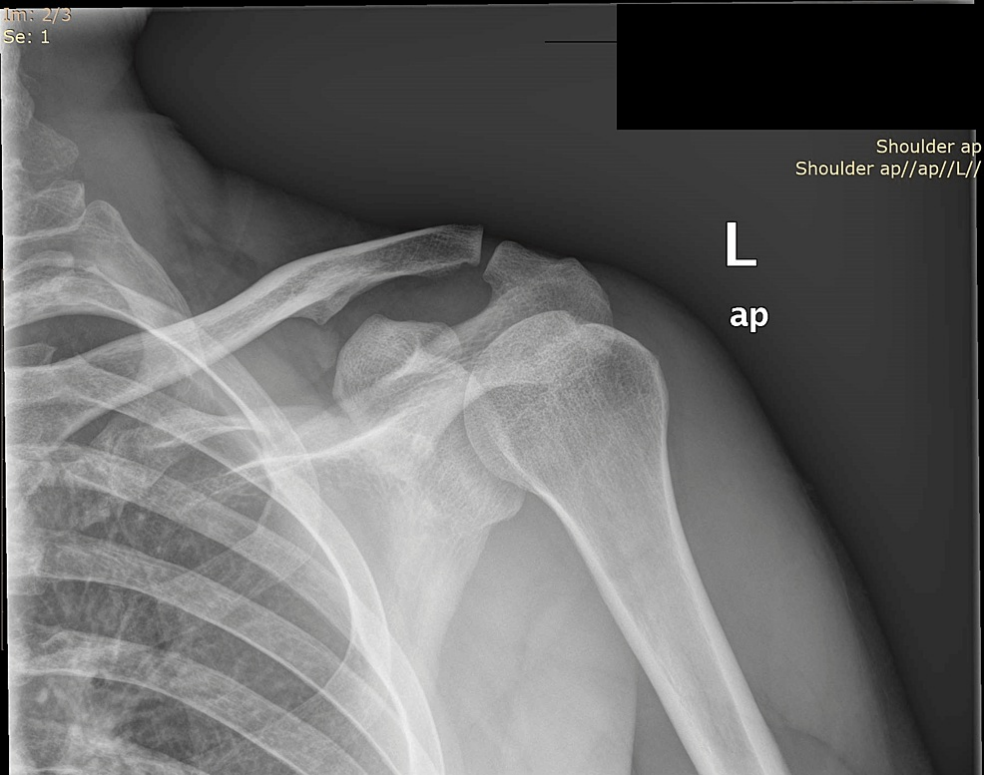
Anteroposterior X-ray of the left shoulder after close reduction by two-step technique maneuver

The reduction was confirmed by repeat anteroposterior radiographs. No fractures were reported on the post-reduction X-rays (Figures 4, 5).

The patient was given instructions and discharged from the ED department, while orthopedic consultation was obtained for a follow-up examination at the orthopedic outpatient department. To date, years after the initial injury, the patient has fully recovered and has gained full range of motion on both shoulders without any signs of instability. No other imaging, apart from plain X-rays, was considered necessary during this period due to the absence of fractures and signs of rotator cuff injury. Written consent was given by the patient for publishing this paper in one of his consultations.

## Discussion

Luxatio erecta can occur in every age group. The two main mechanisms of injury are described as follows: the first mechanism involves an axial compression through a fully abducted arm which drives the humeral shaft through the inferior glenohumeral ligaments, while the second is associated with a hyperabduction stress that directs the humeral neck superiorly against the acromion process and forces the head out of the glenoid fossa inferiorly [3,10]. Consequently, the humerus is locked between 110° and 160° of adduction. The clinical and radiographical presentation is typical among patients with luxatio erecta. The elbow is flexed, the forearm is pronated, and the hand of the patient is either lying on the forehead or behind the head [10]. The humerus is held in the erect position by the pectoralis major, and the humeral head is pulled inferiorly by the teres major and latissimus dorsi. On a typical presentation on an anteroposterior shoulder X-ray, the humeral shaft is parallel to the spine of the scapula on the radiographs, while in anterior or anteroinferior subcoracoid dislocations, the shaft is parallel with the chest wall.

Among all the mechanisms of injury, sport-related ones account for around 26% [5], but the case presented is thought to be interesting, taking into account how the social distancing restrictions during the COVID-19 pandemic influenced training habits and safety. As the use of sports and weight-bearing equipment is becoming increasingly popular at home, gym users should be aware of the dangers of the equipment or should be under the guidance of experts so these types of injuries and the associated complications to be avoided. The efficient treatment of the patient involves stabilization of other concomitant injuries, clinical evaluation of the neurovascular status, and radiological documentation (standard radiographs including an anteroposterior scapular view and an axillary or a lateral scapular Y view). Afterward, prompt reduction with adequate analgesics or anesthesia is thought to be associated with good clinical outcomes. Post-reduction radiographs should be obtained, and in suspicion of a fracture, a CT scanning should be ordered.

## Conclusions

In this article, we present a rare case of TBLE during the lockdown period of the second pandemic wave of COVID-19. Bilateral cases of LE are associated with a high rate of complications (higher than unilateral). ED physicians should maintain an increased awareness for prompt recognition, taking into account the mechanism of injury, the distinctive signs and clinical presentation of this dislocation (a hyperabducted and above the head locked arm), and pitfalls in diagnosis and management must be avoided. The early recognition, followed by prompt, carefully performed reduction, is crucial in avoiding soft tissue injuries (including vascular or brachial plexus injuries).

To our minds, the key in these kinds of injuries is firstly to understand the mechanism of injury and recognize the possible complications with a meticulously performed clinical evaluation of the patient and secondly to perform an uneventful reduction followed by clinical and radiological evaluation (including evaluation the neurovascular status and radiological confirmation).

Great care should be given to polytrauma patients upon their arrival at the hospital. LE is relatively easy to diagnose given the unmistakable arm positioning, but the presence of this injury increases the complexity of managing this type of patient in the ED due to the abducted arms.
